# Spotlight on MicroPulse Laser Trabeculoplasty in Open-Angle Glaucoma: What’s on? A Review of the Literature

**DOI:** 10.3390/vision6010008

**Published:** 2022-01-21

**Authors:** Gloria Gambini, Matteo Mario Carlà, Tomaso Caporossi, Umberto De Vico, Alfonso Savastano, Antonio Baldascino, Clara Rizzo, Raphael Kilian, Stanislao Rizzo

**Affiliations:** 1 Ophthalmology Unit, “Fondazione Policlinico Universitario A. Gemelli IRCCS”, 00168 Rome, Italy; tomaso.caporossi@gmail.com (T.C.); umbertodevico@gmail.com (U.D.V.); alfonso.savastano@policlinicogemelli.it (A.S.); antonio.baldascino@policlinicogemelli.it (A.B.); stanislao.rizzo@gmail.com (S.R.); 2 Ophthalmology Unit, Catholic University “Sacro Cuore”, 00168 Rome, Italy; 3 Ophthalmology, Department of Surgical, Medical and Molecular Pathology and Critical Care Medicine, University of Pisa, 56126 Pisa, Italy; clararizzo2@gmail.com; 4 Ophthalmology Unit, University of Verona, 37134 Verona, Italy; raphaelkilian8@yahoo.it

**Keywords:** glaucoma, laser trabeculoplasty, micropulse trabeculoplasty, micropulse laser, subthreshold laser, micropulse diode laser trabeculoplasty, MDLT, selective laser trabeculoplasty

## Abstract

Glaucoma is the most common cause of permanent blindness in the world, caused by a progressive optic neuropathy. Patients with glaucoma are often treated with topical medicines therapy in order to reduce intra-ocular pressure (IOP). On the other hand, laser therapies, with the introduction of Argon Laser Trabeculoplasty (ALT) and successively with Selective Laser Trabeculoplasty (SLT), were reported to be effective in IOP control, with low adverse effect rates. In recent years, the micropulse laser, a subthreshold laser technology, was introduced with the goal of reducing side effects while maintaining the effectiveness of the laser treatments. Several studies focused on Micropulse Diode Laser Trabeculoplasty (MDLT) in open-angle glaucoma, to evaluate its effectiveness and possible side effects. Promising results were reported, but irradiation circumstances have not been standardized yet and its role as a substitute for previous laser techniques has yet to be defined. As a result, the goal of this review was to analyze the physical principles at the basis of MDLT and to frame it in the open-angle glaucoma management setting, highlighting the advantages and shortfalls of this technique.

## 1. Introduction

Glaucoma is a progressive optic neuropathy, which has become the biggest cause of permanent blindness across the globe [1]. In primary open-angle glaucoma (POAG), the main goal of treatment is to reduce intraocular pressure (IOP) in order to slow the progression of visual field and retinal nerve fiber layer degeneration. To lessen the risk of surgical complications, topical antiglaucoma medicines are frequently used as a first-line therapy before surgery [2].

The first description of laser utilization directed to the trabeculum dates back to 1979, with the introduction of argon laser trabeculoplasty (ALT) [3]. Following that, in 1995 the selective laser trabeculoplasty (SLT) saw the light [4,5]. Since then, they have become widely used for the treatment of open-angle glaucoma (OAG) and have been demonstrated to have a similar efficacy for reducing IOP as topical medications while avoiding their side effects [6]. Several investigations have shown that these two techniques are equally effective. SLT, on the other hand, has the benefit of not leaving a scar on the trabeculum and being repeatable [5,7,8,9,10]. SLT is a noninvasive procedure that delivers energy to pigmented cells of the trabecular meshwork (TM), resulting in the disruption of the pigmented TM endothelial cells, as seen by electron microscopy [7]. In addition to biological alterations, it is believed that this cellular damage results in an enhanced inflammatory response, which in turn causes an increase in aqueous outflow via the TM [11,12]. Transient intraocular pressure (IOP) spikes and ocular pain might occur as a result of the inflammatory response and early pigment dispersion, among other things [11].

Pankratov et al. first published the use of a micropulse laser for retinal photocoagulation in 1990 [13]. Since then, several investigations have proved that stimulation of the retinal pigmented epithelium may be accomplished without causing irreversible heat damage to the tissue [10,14,15]. This technology, which uses a duty cycle algorithm to administer subthreshold therapy to ocular tissues without causing scarring, gained approval for the treatment of macular edema in diabetic retinopathy, retinal vein occlusion, and central serous chorioretinopathy [16,17,18].

Researchers have tested this technique on pigmented trabecular meshwork to see if it may lower intraocular pressure (IOP), increasing the permeability of TM, without causing thermal damage like argon laser therapy does [7]. This notion was backed by a histologic analysis, which confirmed that the micropulse laser did not cause any trabecular meshwork damage [19]. Ingvoldstad et al. presented the first clinical results of micropulse diode laser trabeculoplasty (MDLT) at the annual conference of the Association for Research in Vision and Ophthalmology in 2005 [20]. Since then, a few more studies have studied MDLT using a variety of laser wavelengths and treatment intensities, with varying degrees of success [21,22,23,24,25,26].

Micropulse diode laser trabeculoplasty (MDLT) is a subthreshold laser technique that divides a continuous laser into short on-and-off pulses to allow for cooling between treatments. Cooling the TM during the interpulse phase avoids cellular or morphologic alterations [12]. When compared to ALT, SLT and MDLT did not leave any morphologic alterations to the TM in a research on human corneoscleral rim tissue from cadaver eyes treated with laser trabeculoplasty [19]. The pulse energy can be titrated during SLT until occasional bubble production is visible. MDLT is difficult to titrate since there is no apparent trace of therapy during the surgery, but it significantly lowers post-operative inflammation, pain, and IOP spikes when compared to SLT [27,28].

This promising technology has shown comparable results in terms of IOP reduction to SLT [22,27,28]. However, concerns regarding IOP inter-operator variability have arisen because of the lack of observable treatment endpoints with MDLT, such as the bubbles seen in the SLT technique. Because earlier investigations have shown contradictory findings and irradiation circumstances are not uniform, the efficiency of MDLT requires additional confirmation [27,28,29,30].

As a result, we analyzed the pre-existing literature to discover whether this new technique may offer promising results in IOP management. We conducted research on original articles including the following keywords: “micropulse laser trabeculoplasty”, “micropulse diode laser trabeculoplasty”, “micropulse trabeculoplasty”. We focused on MDLT physical effects and described laser settings. Moreover, original research, which assessed MDLT results on IOP management and possible adverse effects were included, with an emphasis on the comparison between MDLT and SLT.

Since this novel approach may offer a new way to reduce topical drugs burden for glaucoma patients while maintaining a good safety profile, this review aimed to evaluate MDLT results in clinical practice and define whether it can merge with other glaucoma treatments.

## 2. Laser Trabeculoplasty

Laser application to treat the trabecular meshwork (TM) had the goal of enhancing aqueous outflow and thereby reducing IOP [31]. The first description of a trabecular meshwork perforation goes back to Krasnov, who used a ruby laser [32]. A novel non-penetrating trabeculoplasty approach using an argon green laser (major peaks at 488 nm and 514 nm) was published by Wise and Witter in 1979 and became the pilot research and standard technique of argon laser trabeculoplasty (ALT) at the time [3].

During the ALT procedure, the spot size is set to 50 um with a period of 0.1 s and a power range of 400 to 1000 mW. The Ritch lens or Goldmann 3-mirror goniolens is used to determine angle structures. The intersection of the pigmented and non-pigmented TM is burned. The laser power is titrated to generate a tiny bubble or blanching at the laser application location. Different approaches have been proposed: some studies claimed a 180° treatment with 50 TM burns, while others prefer 100 burns over 360° [33,34]. Treating 360° is associated with a higher incidence of pressure spikes, but an additional 180 degrees of treatment can be performed later when a treatment response is appreciated with an initial treatment. The ALT procedure can also be performed with a diode laser. In this case, typical settings are 75-micron spot size, 0.1-s duration, and 600–1000 mW power.

This procedure was nevertheless associated with some adverse events—acute or late IOP spikes—especially in pseudoexfoliative glaucoma, peripheral anterior synechiae and rare cases of endothelial corneal decompensation were reported, despite its effectiveness in controlling IOP [35,36,37,38,39]. Moreover, the IOP-lowering impact of ALT decreased with time and was less noticeable in eyes that had previously failed to respond to ALT [11]. Thermal damage on the structures located near the pigmented TM cells has been cited as a possible cause of ALT problems. As a result, Latina and Park devised the selective laser trabeculoplasty (SLT) approach in 1995, which exclusively targets pigmented cells [4].

In the SLT technique, a frequency-doubled, Q-switched, 532-nm Nd:YAG laser is used [5]. With an energy of 0.4–1.2 mJ, 400 um diameter, confluent spot size, the laser is focused on the TM and 3 ns laser pulses are shot. The amount of energy utilized is titrated to the degree of trabecular pigmentation, with less energy being required as the pigmentation increases. Starting at 0.6 mJ, increase by 0.1 mJ until cavitation bubbles (“champagne bubbles”) occur at a certain point, where the laser energy is lowered until bubble production disappears, defining the level at which the therapy continues. Typically, 100 spots (25 each quadrant) are lasered around the whole TM. Two quadrants (180°) are lasered initially in patients with a strongly pigmented TM, with the remaining two quadrants held in reserve for additional lasering if necessary. The laser effect allows for selective photo-thermolysis and prevents heat transmission to nonpigmented cells in the surrounding area, indeed minimizing injury [40,41]. The thermal relaxation period of melanin in pigmented cells is in fact substantially longer than the 3 ns laser pulse duration of SLT [4]. The most common complications of laser trabeculoplasty are transient mild redness, acute iritis and an increase in intraocular pressure (IOP) in the first week, depending on applied energy. More uncommon complications, such as transient corneal thinning, endothelial decompensation, peripheral anterior synechiae, corneal haze and cystoid macular edema, can occur [42,43,44]. Generally, SLT results are comparable to ALT, with a lower incidence of adverse effects than ALT. Moreover, it has the extra benefit of no scarring of the TM, allowing for follow-up repeatability [43,45].

Concerning long-period IOP stabilization, SLT could not overcome the problem of reduced effects reported in ALT, with Lai et al. indicating a decline in the SLT success rate from 71% at one year to 25% after five years [46]. Some studies hypothesized that TM cellular damage and excessive early pigment dispersion, which leads to high levels of inflammation, are the underlying causes of SLT’s complications. At the same time, SLT-induced inflammation is the main trigger for the increased aqueous outflow through the TM extracellular matrix, which consequently leads to IOP-lowering effects [47,48,49].

Laser trabeculoplasty gained a primary role in POAG therapy setting, not only decreasing IOP but also delaying visual field degeneration, according to many multicenter randomized studies including the Early Manifest Glaucoma Trial and the Advanced Glaucoma Intervention Study, Glaucoma Laser Trial (GLT) [50]. Patients treated with ALT had lower IOP if compared to medical therapy after seven years in the GLT follow-up trial [50].

Moreover, according to the results of the recently published large-scale randomized controlled trial of SLT versus eye drops for first-line treatment of glaucoma and ocular hypertension patients (LiGHT trial), three-quarters of treatment-naive patients who received SLT as a primary treatment achieved their target IOP while remaining drop-free within the first three years of the trial [51]. As a result, laser trabeculoplasty—particularly SLT for its limited damage to TM cells—is currently employed as a first-line therapy for individuals with POAG and ocular hypertension (OHT).

## 3. MicroPulse Diode Laser Trabeculoplasty

In order to limit adverse effects following laser trabeculoplasty, researchers tried lowering the amount of energy provided by each SLT session and discovered that subthreshold energy SLT might be as effective as standard SLT [52,53]. Other types of subthreshold lasers were thus hypothesized to have IOP-lowering potential. This led to the introduction, in 2005, of the Micropulse Diode Laser Trabeculoplasty (MDLT), thanks to the studies of Ingvoldstad and Willoughby [20]. MDLT significantly reduces IOP in patients with primary open angle glaucoma to the same degree as ALT, in the meantime avoiding both thermal damage (as happened with ALT) and cellular damage (the main complication of SLT). 

Micropulse laser safety relies on the fact that the interpulse period allows the temperature of the pigmented cells to return to baseline before the next micropulse, thus preventing cellular damage from cumulative thermal rise or spreading, scarring or morphological changes in the TM tissue. Its mechanism of action is able to avoid post-treatment IOP spikes, even in heavily pigmented TM [19,20,54]. Nevertheless, the molecular effect of the micropulse laser is unknown, and only hypotheses exist. Researchers theorized that ALT, SLT and MDLT act in different ways to obtain the same cellular reaction. In fact, laser trabeculoplasty procedures with diverse laser settings and end points consistently exhibited equivalent IOP-lowering outcomes [21,55].

The principle of newer versions of laser trabeculoplasty aims to increase aqueous outflow while decreasing tissue damage by stimulating a cellular biochemical cascade via cytokine release [55,56]. MDLT’s lower level of laser energy is enough to trigger this therapeutic pathway in live TM cells without the severe coagulative and cellular damage seen with ALT’s and SLT’s greater levels of energy [7,56,57].

### MDLT Technique

MDLT is a low-irradiance therapy that employs a diode laser with a large spot size that generates repeating, short laser pulses in 200-ms bursts, with a 1.7-ms gap between micropulses [20]. It is an adaptation of micropulse technology, in which the duty cycle is the percentage of time that the laser will be active during the treatment duration. This allows the tissue to cool down and prevent evident coagulative damage before the next laser application. On the other hand, the absence of a visible effect on the TM makes the titration of energy difficult.

The first MDLT pilot study was carried out by Ingvoldstad and Willoughby with the IRIS Medical OcuLight SLx 810 nm diode ruby laser system (IRIDEX Corporation, Mountain View, CA, USA). For the procedure, they set a 2000 mW power, 300 um spot size diameter, and 200 ms duration with 15% duty cycle over the full 360° of the TM [20]. Since its introduction, different researchers tried to optimize laser settings and geographical objectives. The classical 810-nm diode laser MDLT approach, claimed in the first years by many investigators, [21,22,23,24] recently gave way to new approaches with 532- or 577-nm diode laser [26,27,28,29,54,58,59,60,61]. The settings for both the 577-nm yellow laser (IRIDEX Corporation, Mountain View, CA, USA) and the 532-nm green laser (IRIDEX Corporation, Mountain View, CA, USA) were generally repeated among researchers, with 1000 mW of power, 300 ms duration, 300 μm spot size, and a 15% duty cycle. However, varying laser settings have been proposed, focusing in particular on the treatment area of trabecular meshwork, as can be seen in Table 1, which underlines the fact that no standard protocol for MDLT treatment has been defined yet. 

## 4. MDLT for Primary Open-Angle Glaucoma

Patients with open-angle glaucoma that was not managed by topical drugs alone were the focus of MDLT trials. The majority of the studies included all kinds of open-angle glaucoma, with only a few of them specifically targeting primary open-angle glaucoma, [23,29,54] and only one study regarding exclusively secondary open-angle glaucoma [30].

The MDLT approach for primary open-angle glaucoma (POAG) was analyzed by Babalola et al. in a retrospective review of Nigerian patients [23]. The main indication for the treatment was an unsatisfactory response to medical management or a reluctance to undergo trabeculectomy. They experimented with a 180° inferior 810-nm MDLT with a 1000 mW power and divided the 30 eyes into three subgroups of 75, 125, and 200 um spot widths. MDLT resulted in a statistically significant initial IOP decrease of 17.2%, which was sustained over a range of weeks to months. Interestingly, they also discovered that the highest spot size, 200 um, was linked to much higher IOP decreases than 75 or 125 um. There was no evidence of significant inflammatory response or impact on visual outcomes [23].

A novel approach was proposed by Abouhussein et al. in an interventional investigation [54]. They introduced the 577 nm diode laser over the 810 nm diode laser used by Ingvoldstad, because the 577 nm allows for larger 300 um spot size. In a six-month follow-up period of 30 eyes, the IOP reduction was found to stabilize on a 21.6% decrease from baseline [54].

Recently, Hong et al. reported a series of 72 Chinese POAG patients who underwent MDLT, using a 532-nm laser system for a 360° angle treatment. In this cohort, 19 cases were initial POAG patients without any medication [29]. The mean IOP before MLT was 20.6 mmHg, dropped considerably following MLT and remained steady over the next 24 weeks reaching an average of 16.5 mmHg. Moreover, no IOP spikes were recorded and the number of glaucoma drugs was significantly reduced from 1.7 to 1.5 per patient, implying that MDLT is a safe and successful procedure in the treatment of POAG [29].

Those studies highlighted the efficacy of the MDLT approach for some POAG patients, with an IOP reduction calculated at about 20 percent from baseline. Nevertheless, it was unknown whether different laser settings or geographical parameters (180° vs. 360° trabeculoplasty) may influence the MDLT effect, and how long the treatment effect would last over the 6-month follow-up period and if it could be repeatable.

## 5. MDLT for Secondary Open-Angle Glaucoma

Makri et al.’s prospective study only evaluated the effectiveness of a single session of 360° 532-nm MDLT in a specific setting of secondary OAG, in particular in patients with pseudoexfoliation glaucoma (PEXG) under prostaglandine analogue monotherapy with ineffective IOP management [30]. When compared to baseline, MDLT treatment resulted in considerably reduced IOP at 1, 3, 6, and 12 months. Globally, about 52% successful rates were reported, with an IOP decrease by 20% or more as compared to baseline. 15% of all eyes were regarded failures because they needed further treatment [30]. This research highlighted the prominent role of micropulse trabeculoplasty as an effective method to lower IOP in patients with secondary OAG.

## 6. MDLT for All Kinds of Open-Angle Glaucoma

Several studies claimed the role of MDLT in open-angle glaucoma management with large inclusion criteria, gathering both primary-angle glaucoma (POAG), normal tension glaucoma (NTG), pigmentary glaucoma or pseudoexfoliative glaucoma. The obtained results were compared with pre-existing approaches, such as ALT or SLT.

### 6.1. 810-nm MDLT

Ingvoldstad et al., in their pilot study with an 810-nm laser, reported IOP reductions of 18.3% with a 3-month follow up. In their approach, laser settings were 2000 mW power, 300 µm spot size diameter, and 200 ms duration on all 360° of TM [20]. Detry-Morel and colleagues investigated the use of MDLT for phakic open-angle glaucoma in 31 eyes of 26 phakic glaucoma patients, carrying out the first prospective randomized investigation to compare MDLT to ALT [22]. The researchers used the identical laser settings as the Ingvoldstad trial but targeted the inferior 180° of TM. Their results were far from resounding: while both techniques were able to significantly lower IOP at the 3-month follow-up visit, MDLT resulted in a significantly lower IOP decrease than ALT (12.2% decrease with MDLT vs. 21.8% with ALT), implying that MDLT was still inferior to ALT in terms of IOP-lowering effects in a mid-term follow-up [22].

In those years, different investigations took place utilizing the same 810-nm laser, but with novel settings. Two investigations, by Fea and colleagues and by Rantala and Valimaki, examined 180° MDLT for open-angle glaucoma instead of the original 360° [21,24]. Differently from the experiments of Ingvoldstad and Detry-Morel, Fea and colleagues’ prospective research employed a smaller spot size of 200 µm, with a 200 ms duration and 2000 mW power on the inferior 180° of TM. The group of 15 eyes showed a substantial post-treatment IOP decline of 22.1% at their 12-month follow-up, while five eyes were not responsive [21]. On the other side, after 19 months of follow up, Rantala and Valimaki reported no mean IOP decline in 40 eyes of 29 patients utilizing similar laser settings, but with a bigger spot size of 300 µm directed to the inferior 180° of TM [7,24]. The IOP reduction of at least 20% was reached only in 2.5% of all eyes at 3-month follow-up, and only 7.5% of their subjects achieved an IOP reduction of at least 3 mmHg. The lack of results in this study was thought to depend on poor laser settings or erroneous targeting of only 180° of the TM. A secondary IOP-lowering surgery was indeed required in half of all patients in the cohort [24].

### 6.2. 577-nm MDLT

Those checkered results offered by the 810-nm laser approach led to the research of novel techniques for micropulse trabeculoplasty. Chudoba et al., in 2014, conducted a pilot investigation introducing the 577-nm yellow laser approach for MDLT on the inferior 180° TM. In this group, the IOP lowering targets were unmet, with a decline of 1.7 mmHg in treated eyes, while in the untreated eyes, IOP decreased by 1.8 mmHg [25].

In 2015, Lee et al. performed a prospective investigation on 48 open-angle glaucoma eyes utilizing a 577 nm diode laser in a 360° MDLT method [26]. Laser settings were 1000 mW power, 300 µm spot sizes and 300 ms treatment duration. They found out a significant reduction in both post-treatment IOP, with a 19.5% average decrease at the 6-month follow up, and IOP-lowering medications. Similar to Fea et al.’s findings [21], MDLT was successful in 73% of the eyes that underwent therapy, with no further treatments needed, and only 7.5% of the eyes experienced a moderate self-limiting anterior chamber response. Moreover, no difference in treatment effects between primary open-angle glaucoma and normal-tension glaucoma was appreciated, and visual acuity during follow up was unaffected [26]. The larger IOP decrease in Lee’s research was thought to depend on the MDLT wavelength (577 nm) being closer to that of SLT (532 nm), since the 810-nm MDLT laser could not be sufficiently targeting the pigmented TM cells [26].

Recently, a retrospective study by Kakihara et al. focused on 577-nm MDLT treatment for OAG patients under maximal tolerable glaucoma eyedrops, to assess the survival rate after MDLT [61]. Failure was defined as a reduction <20% in IOP from baseline or >21 mm Hg during two consecutive follow-up visits or the need for surgical intervention. The efficacy of MDLT in eyes with advanced glaucoma was shown to be restricted in this investigation, since the success rate at 6 months following surgery was only 44%. The authors hypothesized that the optimal MDLT approach may need a steeper learning curve, because higher survival rates were reported when MDLT procedure was carried out by a glaucoma specialist with sufficient MDLT experience [61].

### 6.3. 577-nm MDLT vs. SLT

In recent years, micropulse trabeculoplasty effectiveness was indeed compared to the SLT approach. De Leon and colleagues analyzed the efficacy of 360° MDLT with 577-nm laser, when compared to 180° SLT, in a clinical trial [58]. They treated 121 eyes of 77 patients with open-angle glaucoma, demonstrating that both techniques were able to lead to a significant average IOP drop at the final 3-month post-operative follow-up (19.9% IOP decrease for MDLT vs. 19.8% for SLT). In addition, they also discovered no reduction in drugs used before and after treatment for both groups, which was different from what was evidenced in other studies [26,58,62]. 

As a confirmation of the efficacy of this approach, Abramowitz et al. conducted a trial to compare 360° MDLT with 360° SLT technique. They included 69 patients, all laser trabeculoplasty candidates with open-angle glaucoma on maximally tolerated medical therapy with the need for additional IOP lowering and followed them for 1 year after treatment [28]. Both treatments revealed comparable effectiveness, with MDLT having better success rates than other previously reported trials in reaching the target of a >3 mmHg IOP reduction or a >20% decrease in IOP from baseline values. Moreover, Abramowitz et al. were the first to assess a statistically significantly lower experienced pain both during and after the treatment in the MDLT group, when compared to SLT [28]. During the follow-up period, patients in each group were also examined to identify the percentage of patients who needed further glaucoma treatment, defined as treatment failure. Despite the fact that the MDLT group had a higher failure rate (23.7%) than the SLT group (12.9%), the analysis did not reach statistical significance [28].

### 6.4. 532-nm MDLT

In the last years, a novel approach emerged, with the 532-nm green laser (Iridex Corporation, Mountain View, CA, USA), which took place for micropulse trabeculoplasty. Gossage and colleagues published 2-year results after treatment with 532 nm MDLT in 18 POAG eyes. The laser intensities were set to 300 mW, then 700 mW, and finally 1000 mW. Those who were given 1000 mW had much more superior results, with an IOP drop of 24% after 24 months, confirming the effectiveness of the settings used in the aforementioned studies [63].

In a retrospective study of 2018, 30 glaucomatous eyes underwent a 360° MDLT with 532-nm laser [59]. The authors claimed that the highest IOP decrease was reached on the first day, with a post-laser median early drop of 2 mmHg. The average IOP reduction at the last follow up was 17.9%, with a trend toward larger IOP decrease with higher baseline IOP. Nevertheless, the loss of nearly half the patients at the 19-month follow up and the small sample size were great limitations of that research [59].

### 6.5. 532-nm MDLT vs. SLT

Further studies were conducted to assure 532-nm MDLT effectiveness, against pre-existing approaches. Nevertheless, there is still a lack of long-term data comparing MDLT and SLT with the same wavelength. In 2019, Hirabayashi et al. carried on a comparison among 50 eyes who received 532-nm MDLT and 50 eyes who received SLT, both over 360° of angle per procedure [27]. At six months, the overall success rates for both MDLT and SLT were about 40%, and they were similar in both groups, with the influence of baseline IOP on successful outcome shown to be more strongly connected in the SLT group than in the MLT group. In contrast to SLT, MLT demonstrated equivalent effectiveness regardless of the age of the patients [27]. These results were comparable to those claimed by Abramowitz et al. with 577-nm MDLT [28]. In this research, predictors of effectiveness and safety in MDLT were compared to SLT [64]. Surprisingly, neither the severity of glaucoma nor the pigmentation grade of the TM predicted a positive result in either group, while the only aspect linked with a favorable MDLT result was a lower number of laser shots, suggesting the existence of a threshold in the quantity of total energy necessary for micropulse trabeculoplasty [27].

Sun et al. recently reported a retrospective, comparative cohort study, in which patients with open-angle glaucoma receiving their first treatment of laser trabeculoplasty were included [60]. SLT resulted in an average higher IOP decrease at the 1-month visit, compared to MDLT (16.5 vs. 17.9 mmHg mean IOP), and this decrease in IOP was sustained in the SLT group for a year. When comparing SLT to MDLT, there was a tendency toward a higher decrease in IOP at one year with SLT, with a lower number of glaucoma drugs than the MDLT group, but success rates were statistically comparable in both groups. On the other side, MDLT had lower post-laser IOP rises and less post-operative inflammation than SLT (5% vs. 16%) [60].

A review of the results reported by different studies is highlighted in Table 2.

## 7. Post-Procedure Complications

Very few side effects have been reported after MDLT, enforcing its optimal safety profile. The micropulse laser indeed generates no macroscopic or microscopic alterations to the TM cells, resulting in reduced inflammation and adverse effects. Prior approaches had reported complications: ALT, on one hand, causes coagulative and structural damage to the TM, whereas SLT causes tiny pigment granule breaking, with biochemical damage to the pigmented TM cells [19]. In the MDLT approach, the interpulse period of cooling prevents cellular or morphologic changes to the TM, thus suggesting that interoperator variability of the treatment impact might be an issue since there are no apparent treatment end points, such as “champagne bubbles” for SLT [12].

There were no major, sight-threatening, or non-transient problems recorded in any of the MDLT investigations. The majority of the studies analyzed reported no adverse reactions at all. Visible laser flashes, changes in visual acuity, anterior segment inflammation, and peripheral anterior synechiae, priorly reported after ALT or SLT, were very limited after MDLT. Only a few studies reported transient IOP spikes, defined as IOP increase ≥5 mmHg in the first hours after treatment [21,22,23,60]. This complication was either self-limited or effectively managed with topical or systemic anti-hypertensive medications in all patients, and the IOP was eventually recovered to at least pretreatment levels. Despite this, SLT treatment itself was associated with a 10% rate of post-operative IOP spikes, as reported by several studies, [26,27,28,59,65], which is a significantly higher value when compared to MDLT.

Lee and colleagues, in their study, reported mild but self-limiting anterior chamber inflammation in 7.5% MDLT cases, without changes in visual acuity [26]. Fea et al., in their cohort, found an IOP spike and anterior chamber flare in one patient with pigmentary glaucoma who underwent MDLT treatment. Nevertheless, the IOP normalized after 3 days with systemic drugs [21].

## 8. Discussion

In recent years, the number of laser trabeculoplasty alternatives for treating open-angle glaucoma has increased. Patients may now benefit from new approaches to lower IOP and lessen the burden of glaucoma medical treatment. When compared to the previous SLT or ALT technologies, Micropulse Diode Laser Trabeculoplasty (MDLT) to be equally effective. 

The first studies carried out with the 810-nm approach showed controversial results, with success rates, defined as an IOP reduction >20% or >3 mmHg from baseline, varying from 7.5% to 60% [20,21,22,24]. Moreover, these studies focused on the inferior 180° approach on the TM, which was abandoned in the next years. These results were also biased by higher pre-operative IOPs (from 20.7 to 25.0 mmHg), which may indicate a delay of treatment and consequent influence on post-operative results [20,21,22,24].

In more recent times, the 577- or 532-nm MDLT approaches over 360° of TM were reported to be successful alternatives to classic laser trabeculoplasty procedures for lowering IOP and decreasing the amount of anti-hypertensive drugs, particularly in individuals with primary open-angle glaucoma. Several studies claimed success rates ranging from 37% to 44%, with final post-operative IOPs from 12.8 to 16.7 mmHg in mid- or long-term follow-ups [26,27,28,29,54,58,59,60,61]. Along with an average IOP reduction varying from 7.2% to 21.6%, MDLT was also shown to have no major or long-term side effects in the analyzed research, highlighting its capacity to limit post-procedural IOP spikes or intra-ocular inflammation [26,27,28,29,54,58,59,60,61]. Moreover, intra-procedural pain was reported to be significantly lower with the MDLT, rather than the SLT approach [28].

Nevertheless, the current MDLT technique still employs a variety of laser settings among different studies, making it impossible to evaluate success and complication rates across papers, even if the most frequent and successful settings seemed to be 1000 mW of power, 300 ms duration, 300 μm spot size, and a 15% duty cycle [26,27,28,29,54,58,59,60,61]. On the other hand, the 180° MDLT approach with 577 and 532-nm wavelengths has demonstrated variable IOP reducing effects, but the comparison with the 360° approach has not been clearly defined yet. Future research is needed to examine different laser settings in order to standardize and enhance the MDLT technology. 

High-power prospective research comparing 577 and 532-nm MDLT to more traditional laser procedures, in particular SLT, is currently lacking, and the majority of the literature consists of retrospective studies of poor statistical power. Despite this, cross-sectional consensus was reported among those studies, showing no statistical difference in success rates between MDLT and SLT, suggesting the efficacy of this emerging, forward-looking technique [27,28,58,60].

## 9. Conclusions

MDLT delivers great advantages over SLT and ALT, especially in patients at higher risk of post-laser adverse effects, such as pressure spikes or intra-ocular inflammations. The hope is that MDLT can prove to have equal efficacy, but an even safer profile, when compared with SLT. Novel kinds of approaches may be proposed to exploit the MDLT technique at its best. For instance, reports of successful MDLT treatment after previous SLT have been reported [66], suggesting that this approach may also maintain its effectiveness in a pre-treated trabecular meshwork. Moreover, as far as we know, no studies have been conducted to analyze the repeatability of this approach over time, in order to stabilize IOP decrease in long follow-ups. Further research is needed to highlight whether successive treatments might still be effective, suggesting that a recurrent MDLT treatment may enhance IOP control over time. Eventually, this novel approach could improve open-angle glaucoma management and reduce anti-hypertensive topical drugs burden in those patients.

## Figures and Tables

**Table 1:** MDLT settings in different studies. TM = Trabecular Meshwork.

Study	Laser WaveLength (nm)	Power (mW)	Spot Size (µm)	Spot Duration (ms)	Duty Cycle	TM Degrees of Treatment (°)
Fea et al. (2008) [21]	810	2000	200	200	15%	Inferior 180
Detry-Morel et al. (2008) [22]	810	2000	300	200	15%	Inferior 180
Rantala et al. (2012) [24]	810	2000	300	200	15%	Inferior 180
Babalola (2015) [23]	810	1000	Varying between 75, 125, 200	200	15%	Inferior 180
Lee et al. (2015) [26]	577	1000	300	300	15%	360
Abouhussein (2016) [54]	577	1000	300	300	15%	360
De Leon et al. (2017) [58]	577	1000	300	300	15%	360
Abramowitz et al. (2018) [28]	577	1000	300	300	15%	360
Kakihara et al. (2020) [61]	577	700–1000	300	300	15%	360
Valera-Cornejo et al. (2018) [59]	532	1000	300	300	15%	360
Hirabayashi et al. (2019) [27]	532	1000	300	300	15%	360
Hong et al. (2019) [29]	532	1000	300	300	15%	360
Sun et al. (2021) [60]	532	1000	300	300	15%	360

**Table 2:** MDLT results in different studies. IOP = Intra-Ocular Pressure.

Study	No. of Eyes	Pre-op IOP (mmHg)	Follow-up	Average IOP Reduction (%)	Final IOP (mmHg)	Success Rate *
Ingvoldstad et al. (2005) [20]	21	24.6	3 months	18.3	20.1	- ^†^
Fea et al. (2008) [21]	20	25.0	12 months	21.3	19.5	60%
Detry-Morel et al. (2008) [22]	16	20.7	5 months	12.2	18.6	36%
Rantala et al. (2012) [24]	40	21.8	12 months	-	-	7.5%
Babalola (2015) [23]	30	18.6	160 days	17.2	15.5	-
Lee et al. (2015) [26]	48	18.5	6 months	19.5	14.9	73%
Abouhussein (2016) [54]	30	18.1	6 months	21.6	14.2	-
De Leon et al. (2017) [58]	29	19.8	3 months	19.9	15.7	48%
Abramowitz et al. (2018) [28]	38	18.3	12 months	14.2	15.7	37%
Kakihara et al. (2020) [61]	42	19.1	6 months	-	13.1	44%
Valera-Cornejo et al. (2018) [59]	30	15.6	19 months	17.9	12.8	41%
Hirabayashi et al. (2019) [27]	50	18.3	6 months	11.5	16.5	44%
Hong et al. (2019) [29]	72	20.6	6 months	19.9	16.5	-
Sun et al. (2021) [60]	43	18.0	12 months	7.2	16.7	12%

* Success rate was defined as a IOP reduction >20% or >3 mmHg from baseline. ^†^ Not reported outcomes are indicated by the symbol -.

## Data Availability

The data that support the findings of this study are available from the corresponding author, MMC, upon reasonable request.
